# The Inoculation of Hogs, with Reference to Erysipelas

**Published:** 1885-10

**Authors:** 

**Affiliations:** Of the Royal Veterinary School, Berlin


### Art. XXVIII.—THE INOCULATION OF HOGS, WITH REFERENCE TO ERYSIPELAS.

BY PROF. DR. SCHUTZ,
Of the Royal Veterinary School, Berlin.

The immense losses which the farmers of this country suffer from swine diseases, especially the Hog Cholera, certainly justify the publication of original investigations which are likely to throw any light upon their causes or nature. The following article, taken from the *Archiv für Thierheilkunde*, sufficiently proves the necessity of every one of our large stock-raising States having at its disposal a properly organized experiment station and biological laboratory, and employing men as state veterinarians suitable for carrying on such researches.

In making this translation the essentials only of the original will be given.

It seems that different parts of Germany also suffer severely from enzöotic diseases in swine; among these the Grand Duchy of Baden, the Minister of which has lately organized a very thorough study of these diseases. The *protective inoculation of Pasteur* having been strongly recommended, it was resolved to give it a trial. The general use of this prophylactic measure would seem at the outset to be met by several hindering contingencies, in that experience has shown that the various races, or breeds of hogs, possess a varying degree of susceptibility to the infections of erysipelas, as well as towards the protective influence of vaccine, which would render it necessary to investigate these conditions so as to adopt the protective material to the different races. We may also assume that climatic and telluric conditions, as well as those of feeding, housing, etc., also exert no inconsiderable influence upon the susceptibility of hogs to these diseases, as well as upon the action of preventive inoculation.

In order to test these questions a committee of scientific men was appointed, and a large number of swine placed at their disposal. Prof. Schutz was detailed by the Imperial Board of Health, at Berlin, to attend these experiments.*

Prof. Lydtin, one of the most exact and accomplished veterinarians of Europe, and known in this country through his work on Tuberculosis, was given charge of the experiments.

The inoculation began in April of this year, and will shortly be published in full, and extracts from the Report will appear in this journal.

The first swine inoculated were at the station at Heidelberg, the inoculation being performed by Dr. Cagny, who had been especially detailed by Pasteur, for the purpose. †

---

* It seems well to call attention to the fact that this very active Board of Health does not, like ours, limit its work to human diseases, but extends its researches to all forms of contagious diseases, well knowing that only by this means can there be any hope of finally coming to any accurate knowledge of the nature of special diseases, as well as recognizing the fact that the wealth of a people is as essential as *their health* to a *healthy nation*.—[Ed.]

† It should be remarked that great as is Pasteur's reputation, he has degenerated from the position a true scientist should assume, not publishing his methods of preparing his vaccine in full, so that others can prepare it, or test his experiments; but has gone into the vaccine supply business, notwithstanding the great liberality with which France has treated him.—[Ed.]

Eight swine, about ten weeks old, were inoculated, each being placed, by Pasteur's special request, with a healthy pig. The temperature and weight of each pig was exactly recorded. The fluid for inoculation was enclosed in glass capillary tubes, one end being hermetically sealed, the other covered with a rubber cap. A fresh supply was daily received from Pasteur's laboratory. The material in the tubes was opalescent, of a yellowish color, and was withdrawn from the tubes by removing the rubber cap by means of a previously sterilized subcutaneous syringe. The animals were inoculated in the subcutaneous tissue of the inner surface of the thigh, each receiving about 3 drops of the fluid: 12 cgrms.

Similar experiments were conducted at different stations over the country.

Prof. Schutz expresses himself as highly gratified, in fact, "that the results greatly exceeded his expectations."

He noticed no great changes in the deportment of the inoculated animals, with the exception of a slight tumefaction around the point of inoculation, and an increase of temperature of about 1° C. The normal temperature of the eight inoculated and eight un-inoculated swine was between 39 and 40° C.

Previous investigations, made in the laboratory of the Imperial Board of Health, with regard to erysipelas in swine, have shown that with this name have been designated *two very different diseases*, both of which assume an enzöotic character in North Germany, in one of these *has always been found a very fine Bacillus*—Erysipelas—and the other, a *larger Bacillus*, that *was only colorable on the ends.* These bacilli are always to be found in the organs and blood of sick or dead animals.*

---

* Salmon, in the Report of the Bureau of Animal Industry, 1884, enters into a polemic against Klein, who asserts *hog cholera to be due to a fine bacillus;* while Salmon claims *it to be due to a Dipolococcus*, and also supports his assertion by quoting that Pasteur has found the same. Here we seem to have the key to the whole story. Salmon's diplococcus is probably this bacteria of Loefler, the spare carrying ends of which seem only to be colorable, the middle not taking the color—while Klein found the fine rod of erysipelas. It is a well-known fact that Pasteur never mastered the art of coloring bacteria, and thereby differentiating them until within the last year. His researches were made some years ago.—[Ed.]

The first of these bacilli resembles much those found in septicæmia of mice, and the last those of fowl cholera. Both of these have been cultivated in meat-infusion gelatine. It is, accordingly, of importance to find out which of these two, ætiologically different diseases causes the losses in Baden as well as in Germany and France, and why not in America?—also from which of them the material supplied by Pasteur proceeds, if we are to obtain protection of swine by inoculation.

In order to ascertain whether it was erysipelas or hog cholera, or both, which afflicted the swine in Baden, I procured either the cadaver, or parts of the same, from swine that had perished from the so-called erysipelas. I found that this disease generally came to eruption in Baden in the month of May; but these assertions cannot always be depended upon.

I made covering glass preparations from the spleen of swine, and found in them large numbers of the previously-mentioned fine rods—bacilli.

Prof. Schutz then inoculated a number of white and the tougher domestic mice with this material; also making June cultivations of the same in gelatine, from which he also inoculated the same kind of animals. In from two to four days—according to the species—the animals all died? The microscopic examination of the blood and organs of these animals proved the same bacilli to exist in them as those with which they had been inoculated. Cultivations from them also gave confirmatory results.

Guinea pigs proved to be insusceptible to infection, though further experiments should be made before this evidence is looked upon as conclusive. Loefler has also shown that they are resistant to the influence of the bacilli of mouse septicæmia, though some local reaction is perceptible.

Rabbits succumbed to inoculation. The bacilli were found in their tissues.

It was now necessary to prove these bacilli upon swine. This was done with material from the organs—spleen—of swine that had succumbed to the natural disease, as well as with June cultivations which had been taken directly from swine, as also from those that had been carried through the mice.

For the full report of these experiments, as well as the microscopical results, we must refer to the original; suffice it to say, each one of the inoculated swine perished from erysipelas as from the natural disease.

It should be remarked that the spleen offers the most favorable locality to examine for the bacteria of this disease, as well as many other diseases, excepting human cholera.

The method of preparing covering glasses for superficial study of these questions, is as follows:

1st. Clean the glass thoroughly.

2d. Take a pair of delicate forceps, and then with a disinfected and clean knife cut quickly into the substance of the organ—(or of fluids the same method, except the cutting, is not necessary); then take up a very little of the organ and spread it out as thinly as possible on the covering glass; as soon as it has dried in the air, take the glass by the edge with the forceps and pass it slowly through the flame of a spirit lamp (Brunsen, if convenient) two or three times; the side on which the material is spread must be uppermost. This procedure coagulates the albumen, and everything adheres firmly to the glass. Then drop upon the glass sufficient of an aqueous solution of gentian violet, or some dark aniline color; let it stand two or three minutes, and then wash off. Dry the glass on filtering or white blotting paper. Place a drop of oil on a slide and put the side of the covering glass containing the material upon it. An ordinary microscope is useless.

To the study of bacteria one must have oil-immersion objectives and an able condensor.

The question now open is: what are the conditions in hog cholera?

Can we not produce an inoculative and preventive material in this country?

Must we sit still, and let all this work be done in Europe, while our hogs are dying almost by the millions?

All that can be said is, if the States will give us a chance the veterinary profession can produce the men to do the work.

### Art. XXIX.—THE ETIOLOGY OF ANTHRAX.*

BY PROF. BOLLINGER AND DR. THEO. KITT, OF MUNICH.

Bollinger concludes from the study of the epidemiology of anthrax made by Dr. Friedrich upon the influences of moisture of the earth and its temperature as well as that of the atmosphere, that an eruption of the disease is favored :

1st.—By a certain moist or swampy nature of the locality. 2d.—By the infection of the earth by the germs of anthrax. 3d.—By the sinking of the ground water. 4th.—By a certain degree of increase in its warmth following on the same.

As the rise and fall of the ground water in any locality is dependant principally on the amount of rainfall and somewhat, also, on the degree of dampness prevailing in the atmosphere, we are thus enabled to judge approximately of the probability of an eruption of anthrax in anthrax regions from the results of meteorological observations.

The prophylactic treatment of such localities by means of drainage, and by avoiding the pollution or infection of the earth with anthrax material—cadavers, the manure or offal of diseased animals, will always be the chief means of preventing an outbreak of the disease.

The investigations made by these observers have conclusively shown that variations in the earth's moisture plays the same *rôle* in anthrax and rinderpest as it does in typhus (typhoid) and cholera. Kitt's investigations have led him to conclude that the alvine discharges from cattle affected with anthrax play the most essential part in the extension and support of the disease ; *i.e.*, dispersion of the virus and infection of the earth. The alkaline or neutral reaction of the fæces of cattle represents, in a certain manner, a natural, solid medium for bacterial cultivation, or the support of germ life in the sense of Koch.

It is only necessary that we place the freely discharged fæces of cattle under a suitable bell-glass to observe that the different varieties of fungoid life each, at first, develope as isolated colonies, or pure cultivations. In spontaneous cases of anthrax, *i.e.*, such as find their origin in spore-infection, and not

* "Deutsche Medicinische Wochenschift," 1885. No. 29, p. 507.

by inoculation, we always find the fæcal discharges more or less hæmorrhagic and covered or mixed with mucous, having a decidedly alkaline reaction.

It has also been shown that in spore-infection the chymus contains innumerable numbers of the anthrax germ in the form of rods, threads and spores. We are thus justified in concluding that the inficiens of anthrax is plentifully mixed with the fæces in cases of natural infection. When the temperature is suitable—the hot days of August and September—it is probable, as well as possible, that the anthrax inficiens thus contained in the discharges of such patients finds most favorable conditions for the development of permanent spores, thus providing a natural means for the continued infection of localities where such material has been dropped by animals or placed by man.

Having made such observations in the noted anthrax regions of the Bavarian Alps, Kitt was incited to make a series of experiments with the fæces of horses and cows alone, as well as mixed with blood and urine from these and other animals. These materials were first sterilized by exposure to hot steam for about six hours, and then inoculated with anthrax blood and pure cultivations of the germs of the disease. Upon the alkaline fæces from cattle the germs developed to threads and spore-carrying bacilli; remarkably luxuriant cultivations were observed upon the same medium in which blood had been well mixed before sterilization. That the germs developed were in reality those of anthrax was proven by feeding a small quantity of the above medium with the germs on it, to sheep. Even when anthrax blood was mixed with freshly discharged fæces, the germs developed at first as pure cultivations when placed under the bell-glass, but naturally the various colonies of fungi soon ran into one another. Neither fresh horse manure nor that which had been mixed with blood or urine offered very favorable conditions for the development of anthrax bacilli. The natural reaction of horse manure is generally acid.

Neutral or alkaline urine from cattle, horses or sheep did not offer the same favorable conditions for the development of anthrax bacilli that has been found in the neutral or alkaline urine of man.

While the desiccation of such manure is known to be detrimental or finally fatal to the continued vitality of the rod bacillus, it is known that such conditions are without influence upon the spores of this disease.

